# The Hospital Isolation of Infectious Diseases

**Published:** 1913-10-18

**Authors:** H. Franklin Parsons

**Affiliations:** late Assistant M. O., Local Government Board


### THE HOSPITAL ISOLATION OF INFECTIOUS DISEASES.*

### IV. Administrative Difficulties in Small Isolation Hospitals (*continued.*)

By H. FRANKLIN PARSONS, M.D., D.P.H., late Assistant M. O., Local Government Board.

*Rural District; Population* 28,000; 1904.—The isolation hospital is a temporary erection; it comprises two wards of four beds, and, in an adjacent building, living-room, bedroom, and pantry for the caretaker. The whole arrangement is rudimentary, and the hospital is ill-equipped; thus there is no mortuary, no laundry, no disinfecting apparatus, no ambulance, no drainage, no water supply. All the water required has to be carried from a farm about a quarter of a mile distant. Up to the time of the inspector's visit only one patient had been treated in the building. She died in hospital, and this fact has militated against the use of the hospital, having prejudiced the public against it. The medical officer of health acts as medical officer of the hospital, but without salary or remuneration. He lives two and a half to three miles away, and there is no telephonic communication between the hospital and his residence.

*Urban District; Population* 10,000; 1901.—There is a hospital belonging to the Council within the district; the cost of this has been defrayed from time to time out of current rates. The construction and arrangements of this hospital are not such as would meet the requirements of the Board in the case of buildings erected under their sanction. The original buildings consisted of some dog kennels and sheds and outbuildings pertaining to them. The original establishment has been added to from time to time, and the buildings are partly of wood and partly of brick. Quite recently a new brick building has been added as a ward for enteric fever. There is provision for twelve patients at this hospital, but all the wards, the nurses' bedrooms and sitting rooms, and the kitchens are under the same roof, and in direct aerial communication with one another. The building is only suitable for the treatment of one form of infection at a time, but its use has not been restricted in this way. There is no unclimbable fence round the hospital, and there is no purification of the hospital sewage before it is discharged into the river. The District Council have no ambulance, but at times the hand-ambulance belonging to the Guardians is borrowed from the workhouse in order to convey a patient to the isolation hospital.

*Rural District; Population*, 24,000; 1903.—Not any of the cases of diphtheria were removed to hospital. In some instances something in the way of home isolation was attempted; in others the sick child appears to have been nursed beside the fire of the general living-room. In reply to a query of the Board, the District Council wrote that "the infectious hospital was at present empty, and diphtheria and other infectious diseases could be treated there if the medical officer deemed it advisable to move any patients there." But the hospital being unprovided with any building for lodging a caretaker or a nurse, the medical officer has, up to the present, not deemed this advisable, except in the case of a tramp with smallpox, when hasty and inconvenient arrangements had to be made for his admission and treatment. The hospital consists of an iron building of two wards, in each of which eight beds are placed. The cubicle contents of each of these wards is 6,592 feet, giving only 824 feet per bed. The absence of an administration block renders the hospital well-nigh useless.

*Rural District; Population*, 16,000; 1907.—A wood and iron temporary hospital was erected out of current revenue. This hospital was supposed to accommodate twelve beds, and had three wards, as well as nurses' quarters. It was accidentally burnt down during a high gale, and apparently the nurse-matron and her two patients only just managed to escape from the building, which was rapidly burnt to the ground.

*Urban District; Population*, 23,000; 1900.—The isolation hospital comprises two buildings, one of which is an old dilapidated two-storey cottage of seven rooms, of which four are available for patients. The capacity of these several "wards" is

* Previous articles appeared on June 21, July 19, August 9, and September 27.

3,300 cubic feet, 1,220 cubic feet, 1,660 cubic feet, and 848 cubic feet. At the time of the inspector's visit they contained six beds, one bed, three beds, and two beds respectively. Each room has a fireplace and small window, except the smallest, which has no fireplace. The inspector was assured, however, that the latter room had been very seldom used for a patient, but had been more frequently used as a mortuary. The remaining rooms are used as kitchen, scullery, and caretaker's bedroom. Six sufferers from enteric fever were being treated in this unsuitable building on the occasion of the inspector's visit in the two first-mentioned rooms. Attached to this building are two pail-privies, in which all the discharges of patients, mixed with disinfectant, are placed; the contents are removed weekly by the contractor. The drainage is conveyed in pipes to a hollow at the lowest part of the site, close to the roadway; here it accumulates, or soaks gradually into the ground. There is no disinfecting apparatus of any kind. The bedding consisted of common flock or chaff mattresses. The second building was one of corrugated iron, erected to meet a threatened epidemic of smallpox, and contained no patients at the time of visit.

*Urban District; Population*, 22,000; 1899.—There is no isolation hospital in the district, in spite of the reiterated advice of the present medical officer of health and of his predecessor. Many years ago the Council purchased a house adjoining their offices, with a view of using it for isolating persons suffering from infectious diseases, but it has not been used for this purpose. For several years past it has been used as the dwelling of the surveyor.

*Rural District; Population*, 21,000; 1899.—There is no isolation hospital in the district. In 1893 a portable hospital was purchased by the District Council, and was erected on a site within the district under an agreement by which the Council were compelled to remove it at the end of six months. It was afterwards stacked in a shed. Numerous attempts to find a new site for it have since been made, but have always been frustrated on account of opposition in the several localities selected. The hospital is now to be sold.

*Rural District; Population*, 17,000; 1906.—The Rural District Council do not possess an isolation hospital building. They do, however, possess what is called a "portable hospital." This is an ingenious contrivance of numbered wooden sections, which can be put together in a few hours, so as to form a small building, which is supposed to be capable of accommodating two to four patients. A van on wheels is expected to be capable of supplying accommodation for a nurse and for cooking. The internal dimensions of the hospital building are 20 feet by 12 feet, and from 7½ feet to 8½ feet high. These structures, which are stored at the workhouse, were provided in 1901 at a cost of £127. They have never been used. Indeed, the hospital had never been put together since its manufacture until the inquiry took place. It would, in fact, be a serious matter to utilise these structures for the purpose for which it was intended.

The Rural District Council possess a modern steam disinfecting apparatus. It is on wheels, and it was expected that it would be possible to take it from one part of the district to another as required, but its weight is so great that its portability is very limited. (The district is in most parts a very flat one.) Practically it is only used when an outbreak of smallpox occurs, and sometimes after a severe case of scarlatina.

Such examples could be multiplied if necessary. It may perhaps be pleaded in excuse for such unsatisfactory arrangements that the resources of the districts are insufficient for the provision of costly hospital accommodation, but this, as already pointed out, is a reason why neighbouring authorities should co-operate, since in this way satisfactory hospital accommodation may be obtained by each authority at a less cost than by providing a separate hospital.

At hospitals of so rough a description as those above mentioned it is only to be expected that the nursing arrangements should be often unsatisfactory; indeed nursing institutions have sometimes declined to send nurses to hospitals where it was known that there was not proper accommodation for them. But in other instances defective nursing arrangements have been found to exist even at hospitals where reasonably sufficient accommodation existed, owing to want of appreciation on the part of the local authority of the requirements of the sick, to their being unwilling to pay for the continued maintenance of trained nurses at times when the hospital was empty, or to the hospital being overcrowded with patients either during an epidemic or through the practice of the local authority of taking in patients on payment from other districts. The following are some instances of unsatisfactory nursing arrangements reported by the Local Government Board's inspectors:—

*Borough; Population*, 25,000; 1907.—Hospital had been erected and enlarged by means of loans. "Although the enlargement of the administrative block, originally the porter's lodge, afforded accommodation for trained nurses, the Council preferred comparatively recently to act upon their own judgment in this matter, and advertised for untrained women to act as nurses. Two women without any qualifications for the post were appointed to act when required, but they have never acted as nurses owing to the very natural opposition of the medical officer of health, who was not previously consulted in the matter. The hospital is now staffed by two maids and by a caretaker and his wife; trained nurses are engaged as required." [Enteric fever is endemically prevalent in the borough, but only a comparatively small proportion of cases had been removed to hospital.]

*Borough; Population*, 15,000; 1901.—The hospital was an old farm-house, which had been acquired in 1881 as an isolation hospital for the joint use of the urban, rural, and port sanitary authorities. Its sanitary condition was found to be very defective. "A labourer and his wife live on the premises as caretakers, for which they are paid £30 per annum. When there are patients in the hospital the man is paid an extra 15s. per week and the woman is paid 3s. per week for each patient up to a limit of 12s. for acting as nurse. She has not had any hospital training. Hitherto the various authorities concerned have not provided a trained nurse, although private practitioners, who are allowed to attend their own patients in the hospital, have on occasion installed an efficient nurse in charge of such cases. This arrangement has been productive of friction owing to the respective duties of the nurse and caretaker's wife in such instances not having been accurately defined."

*Urban District; Population*, 6,000; 1908.—Hospital erected by means of loan; a well-built structure, comprising a two-ward pavilion, an administration block, and a laundry, etc.

"I found it difficult to obtain any very precise information relative to the administration of the hospital, since there is no medical superintendent to supervise the hospital. The staff at the time of my visit consisted of the matron, who was formerly a nurse at a fever hospital, and a servant, the surveyor being, I gathered, responsible generally for the catering. Patients are attended by their own medical attendant, except when the parents or patients are too poor to pay, in which case arrangements have been made for the attendance of the medical officer of health. The medical officer of health has been requested by the District Council to admit only such cases as cannot be properly isolated at home. In practically all cases some weekly charge is made, the usual charge being about 2s. In some instances the non-payment of this charge has resulted in litigation over rather small sums. The existing administration of the hospital cannot be regarded as at all satisfactory, more especially the arrangement by which patients are treated actually in the administration block."

*Urban District; Population*, 17,000; 1899.—The hospital is a permanent structure erected by means of loan. No patients had been admitted since 1888, when thirty-five cases of smallpox were treated there, although enteric fever had been endemic in the town.

"During the smallpox outbreak the then Local Board issued a tariff setting forth the charges that would be made on account of patients treated at the hospital, and requiring a written guarantee from some responsible person that such charge in each instance would be paid. The charge per week demanded under this tariff, which still exists, varies from 5s. to 10s. for children under five to £1 or £2 for persons of twelve years of age and upwards.

"A caretaker, who is the wife of a water inspector employed by the Council, is in charge of the hospital. For this duty she receives no remuneration, but is allowed 3s. 6d. per week during the winter months to provide fuel for airing the wards. She lives with her husband in the administrative block, and in the kitchen the sheets and blankets for the wards are kept. The interior of the hospital was generally in a clean condition and had been recently painted and whitewashed, but the grounds immediately surrounding the buildings were in a very neglected condition."

In this last instance the non-use of the hospital over a period of more than ten years was evidently the result of the high tariff charged for admission, but even where there is no such hindrance it is not uncommon for an isolation hospital serving a comparatively small district to be without patients for periods of several months together. The problem in the case of such hospitals is to combine on the one hand readiness for use at short notice and efficient administration when in use with, on the other hand, economical maintenance at times when the hospital is void of patients.

A frequent arrangement is to appoint a married couple to live in the hospital as caretakers; if the man has served in the Army Medical Corps or similar position and the woman has had nursing experience, so much the better. Their duties are to maintain the hospital in proper order when empty and to assist in the service when there are patients. The man may then act as porter, messenger, disinfector, ambulance driver, etc., and the woman as housekeeper or cook, or even as nurse if there are only one or two cases of no great severity. If the cases are more numerous or more severe, nurses are engaged from a nursing institution. The drawbacks to this arrangement are that it may be difficult to find a couple both of whom possess suitable qualifications for the post; that unless the wife is a skilled nurse, a patient dangerously ill, admitted on an emergency, may be seriously prejudiced; and that when trained nurses are engaged friction is apt to occur between them and the caretakers, who may be of a somewhat different social class.

On the other hand, to maintain a staff of trained nurses in idleness for long periods may be regarded as a waste of money, and is not likely to conduce to their efficiency when occasion for their services arises. Nevertheless, it is desirable that every hospital, however small, which professes to be kept in readiness to receive patients—and a hospital which is not so kept is of comparatively little use as a protection to the district—should have permanently engaged at least one trained nurse. A middle-aged woman trained as a nurse but who had given up the more active duties of her profession would be suitable as nurse-matron, and her services might perhaps be utilised as district nurse at times when the hospital was empty. Unless married, she should be provided with a servant, as a woman could hadly be expected to live alone in a situation so lonely as that of an isolation hospital. Things requiring a man's attention might be allotted to an officer or non-resident servant of the District Council, and the hospital should have telephonic communication so that assistance could be summoned if necessary. Arrangements under which the matron contracts for the supply of nurses when the hospital is in use or for the feeding of the nurses employed have not been found to work satisfactorily, and are not to be recommended.

